# Experiences and challenges in the management of pregnancy-associated breast cancer at the Korle Bu Teaching Hospital: a review of four cases

**DOI:** 10.3332/ecancer.2020.1140

**Published:** 2020-11-10

**Authors:** Josephine Nsaful, Verna Vanderpuye, Aba Anoa Scott, Florence Dedey, Samuel A Oppong, Rita Appiah-Danquah, Nelson Damale, Benjamin Fenu, Theodore Wordui, Joel Yarney, Joe Nat Clegg-Lamptey

**Affiliations:** 1 University of Ghana Medical School, College of Health Sciences, University of Ghana, Korle-Bu, Accra, GA-379-5258, Ghana; 2 National Centre for Radiotherapy, Oncology and Nuclear Medicine, Korle Bu Teaching Hospital, Accra, GA-379-5258, Ghana; 3 Department of Obstetrics and Gynaecology, Korle Bu Teaching Hospital, Accra, GA-379-5258, Ghana; 4 Department of Surgery, Korle Bu Teaching Hospital, Accra, GA-379-5258, Ghana

**Keywords:** gestational breast cancer, pregnancy-associated breast cancer, multidisciplinary team

## Abstract

Breast cancer is the commonest female cancer worldwide and the most common malignancy during pregnancy. The current management of breast cancer is based on patient and tumour characteristics, preferences and disease stage. In pregnancy-associated breast cancer, the gestational age influences treatment options. Sequencing of therapies is guided by safe imaging options, timing of delivery and prognosis. Systemic therapy options in the neoadjuvant, adjuvant and palliative settings are limited due to safety concerns of the unborn foetus. In resource-constrained regions, the application of safe options may be challenging. This paper reports four of such cases managed in Ghana using a multidisciplinary approach and local resource-appropriate evidence-based practices. Maternal and foetal outcomes were acceptable with none resulting in termination of pregnancy.

## Introduction

Breast cancer is the commonest female cancer worldwide [1]. It is also the common malignancy in pregnancy, found in 20% of pregnant breast cancer patients aged below 30 years [2]. Gestational breast cancer, also known as pregnancy-associated breast cancer (PABC), is breast cancer diagnosed during pregnancy and the first year post-delivery or during lactation [2, 3]. The overall incidence is 0.2%–3.8% and is increasing as more women delay childbirth [3–5]. Current management of breast cancer is guided by patient and tumour characteristics, preferences, and stage of the disease. In pregnancy, the gestational age influences treatment choices, and the goal is the safe delivery of a term baby without worsening the patient’s prognosis. Many of the standard diagnostic investigations, systemic therapies and radiation therapy recommended for breast cancer management are relatively contraindicated in most phases of the gestational period risking foetal exposure to carcinogens [6]. Up until now, the safest chemotherapy agents in pregnancy, Adriamycin, cyclophosphamide and 5 Fluorouracil, administered between 13th and 32nd week of pregnancy [7]. This is a small window of opportunity to actively intervene especially in patients presenting with advanced disease. Other options are deferred until postpartum, unless the benefits outweigh the risk to the foetus [8, 9]. The management of breast cancer in a pregnant woman requires a dedicated multidisciplinary team (MDT) comprising oncologists, surgeons, obstetricians, neonatologist, psychologists, social workers, nurses and most importantly the patient and family to evaluate the risk and benefits of management options [6]. It is not unusual to have suggestions for therapeutic abortion for pregnant women diagnosed with breast cancer in Africa, as local data supporting good outcomes of foetus and patients are limited [10]. There is, however, global data discussing the impact of pregnancy on breast cancer outcomes, and the safe application of therapies that reduce poor foetal outcomes [11, 12].

Poor outcomes in PABC could nonetheless be attributed to late stage at presentation as a result of diagnostic delays, as well as delays and limitations in instituting standard life-saving breast cancers [13, 14].

Access to recommended resources depicted in international guidelines impacts the success of safely managing gestational breast cancer to term. This paper shares some experiences and challenges in the management of pregnancy-associated breast cancer using a multidisciplinary approach in a low- and middle-income country (LMIC).

## Case presentations

### Case 1

A 33-year-old first seen in March 2016 was 8 weeks pregnant and had noticed a painless left breast lump and bloody nipple discharge 5 months prior to presentation. We found a suspicious 6 cm lump and mobile axillary lymph node, clinically staged T_3_N_1_M_0_. Core biopsy confirmed an invasive carcinoma NST (no special type) grade 3, triple-negative. MDT recommended an upfront mastectomy and axillary lymph node dissection followed by chemotherapy (cyclophosphamide, Adriamycin and 5-fluorouracil (CAF)) in the second trimester. Following an uneventful completion of chemotherapy, she was successfully induced at 36 weeks’ gestation and delivered a healthy 2.7 kg female. She then had radiation therapy to the chest wall and supraclavicular region postpartum. At her 4-year follow-up, she had no evidence of disease recurrence and the child had normal developmental milestones.

### Case 2

A 38-year-old presented at 5 months’ gestation in January 2017 with a 2-year history of a right breast lump. Core biopsy confirmed invasive carcinoma NST grade 2, ER /PR negative, HER2 positive. She was staged cT3N1M0_._ She completed 4 cycles of CAF per MDT recommendation at 32 weeks with a partial clinical response (50%). A joint caesarean section and mastectomy with level II axillary lymph node dissection were performed at 36 weeks, delivering a healthy 2.4 kg female. Breastfeeding was prohibited, and she continued chemotherapy 3 weeks after delivery with four cycles of paclitaxel with Trastuzumab followed by chest wall radiation. There was no evidence of disease at a 2-year review. The child has remained healthy.

### Case 3

A 26-year-old woman first presented in April 2018 with a year’s history of a left breast lump and a family history of breast cancer in two first degree relatives. The lump was 8 cm in diameter but had no associated palpable axillary lymph nodes. Unfortunately, she defaulted for 7 months and returned with an extensive locally advanced disease. Core biopsy revealed an invasive carcinoma NST grade 2, ER positive, PR positive, HER2 negative and Ki67 >20%. She again defaulted and returned 2 months later, newly married and 9 weeks pregnant; the lesion now involved the whole breast and was fixed to the chest wall. She developed a pleural effusion within weeks for which she had thoracocentesis and pleurodesis. Now staged T4cN2M1, we commenced CAF chemotherapy at the start of the second trimester (at 14 weeks gestation). She completed all six cycles of palliative chemotherapy (CAF) by 34 weeks’ gestation with partial clinical response and no untoward side effects. Labour was induced at 38 weeks, and she delivered a healthy 2.5 kg female. Breastfeeding was prohibited, and she was started on tamoxifen 20 mg daily. There was no evidence of relapse at 1-year follow-up, and the child remained healthy.

### Case 4

A 33-year-old presented in October 2019 with an ulcerated left breast lump and 16 weeks pregnant. Core biopsy confirmed invasive carcinoma NST grade 3, ER positive, PR negative, HER2 positive and Ki67 >50%. Metastatic workup found liver and lung metastasis and was staged T_4b_N_2_M_1_. MDT recommended six cycles of CAF prior to delivery. Following the fourth cycle, she developed uncontrollable generalised tonic-clonic seizures without any evidence of eclampsia. She became semiconscious, with incoherent speech and nystagmus and SpO_2_ ranged between 90% and 78%. She was started on IV mannitol, dexamethasone, phenytoin and midazolam on suspicion of brain metastases, but attempts at magnetic resonance imaging (MRI) failed due to the uncontrollable seizures. After 24 hours of status epilepticus, her next of kin gave informed consent for emergency caesarean section. Predelivery ultrasound had shown a 33-week foetus with severe intrauterine growth restriction. The mother continued to deteriorate and died 72 hours after surgery. A 1.2 kg male delivered was admitted to a neonatal intensive care unit for 3 weeks due to low birth weight and prematurity. The baby survived and weighed 3.7 kg at 3 months.

## Discussion

We have summarised four cases of Pregnancy-associated breast cancer (PABC) that presented to our facility. The stage at diagnosis ranged from locally advanced to metastatic disease. One patient with poor prognostic features succumbed to the disease, and all four babies are currently alive with no adverse effect reported. None of the patients had a termination of pregnancy. A multidisciplinary team approach is unparalleled in the planning of the most effective treatment sequence to ensure the best outcomes for both mother and baby, especially as therapeutic abortion does not improve outcomes [15].

PABC presents a challenge as the welfare of both mother and foetus is crucial in decision making. There is a paucity of data from large trials on the management of breast cancer during pregnancy, and therefore, clinical evidence for treatment is limited. Many existing guidelines are based on international consensus [15, 16].

The physiological changes in the breast that occur during pregnancy and lactation make the diagnosis of PABC challenging as there may be overlapping signs and symptoms resulting in presentation and diagnostic delays. Many therefore present with locally advanced disease as seen in this case series [9, 17]. This is compounded by myths and misconceptions surrounding breast cancer and its treatment in Africa, contributing to late presentation and noncompliance to timely interventions during pregnancy. In the Ghanaian context, fertility and childbearing is considered essential in society, and the treatment of breast cancer may be seen as a threat.

The majority of PABC have biologically aggressive features, usually invasive ductal carcinoma, grades 2 and 3 [3]. A review of the literature showed triple-negative (oestrogen receptor (ER) negative, progesterone receptor (PR) negative and HER-2 negative) breast cancer to be the most common subtype, followed by the luminal B (ER and/or PR positive and either HER-2 negative or HER-2 positive ) subtype. These categories are known to be aggressive and have been associated with poorer outcomes in PABC [3, 5, 12]. Other poor prognostic factors frequently encountered in PABC are large tumour size, node positivity and advanced stage at diagnosis [6]. However, other factors related to the physiology of pregnancy and the postpartum period such as hormonal mechanisms, extracellular matrix remodelling and lymphangiogenesis inducing tumour cell proliferation may contribute to the poor prognosis of PABC [3, 5, 18, 19]. Observed risk factors associated with PABC are family history, including BRCA mutations, advanced age at first pregnancy and poor previous breastfeeding patterns [20]. A study of 718 cases from Nigeria observed an increased risk in women with high parity contrary to other reports, emphasising the need for regional research [21]. Women with BRCA mutations ideally should be offered genetic and fertility counselling, but this is expensive and limited in many LMIC. There is sufficient data demonstrating no added risk to patient, foetus or breast cancer outcomes in BRCA mutated pregnant women [22].

### Diagnosis

All patients with PABC should undergo triple assessment investigations as recommended in nonpregnant patients, taking precautions to protect the foetus. The Korle Bu Teaching hospital in Accra, Ghana, is a tertiary level facility equipped with enhanced diagnostic procedures including digital and tomosynthesis mammography, MRI, computed tomography scan (CT scan), technetium bone scans and pathology services including immunohistochemistry. Out of pocket payments for many of these investigations remain prohibitive in many low- to middle-class income earners, posing a challenge in the management of PABC.

Mammography with abdominal shielding in pregnancy drastically reduces exposure to the foetus and is therefore not an absolute contraindication [16, 17]. However, the sensitivity of mammography is reduced in pregnancy and postpartum due to higher density and loss of contrasting fat in the pregnant and lactating breast and may limit diagnostic accuracy. Ultrasound scans have negligible radiation exposure and can be substituted for mammography, guide needle biopsy and diagnose liver metastasis [15, 17].

CT scans should be avoided to reduce cumulative radiation exposure to the foetus and may be substituted with chest x-rays with appropriate foetal shielding to safely diagnose lung metastases if required [16, 23].

Magnetic resonance imaging (MRI) is a useful tool for imaging of many body parts with low risk of radiation exposure and recommended in pregnancy. It, however, still requires cautious application in the first trimester [15]. MRI without Gadolinium (which predisposes to systemic fibrosis) is the imaging of choice in diagnosing bone, brain and other metastasis in PABC [24]. In our practise, we tend to rely on ultrasonography as MRI is not readily affordable. Technetium bone scintigraphy and positron emission tomography scans are absolutely contraindicated and must be deferred to the postpartum period [16].

### Treatment

It is recommended that PABC is managed based on the existing guidelines for a nonpregnant patient. However, modifications of systemic agents and sequencing of interventions to protect the foetus and mother while maximising breast cancer outcomes are the priority. Early diagnosis of pregnancy, confirmation of accurate gestational age, patient preferences and factors, as well as specific tumour characteristics, are important factors to consider in treatment planning. Treatment interventions, especially with curative intent, should not be unnecessarily delayed because of pregnancy unless the pregnancy is near term. In nonpregnant breast cancer patients, delaying chemotherapy beyond 4 months is associated with diminished efficacy and the same applies to PABC [25]. Termination of pregnancy is not associated with improved breast cancer outcomes [8].

Surgery is the definitive local treatment for gestational breast cancer presenting resectable disease and appears to be associated with minimal foetal risk at any stage of pregnancy [15, 23]. Sentinel node biopsy in pregnancy is controversial [15]. Mastectomy is the recommendation of choice, but breast-conserving surgery (BCS) may be recommended if radiotherapy can be safely delayed till postpartum. Due to the advanced stage at presentation as in our case series, the majority of PABC in Africa usually require neoadjuvant chemotherapy and mastectomy with very few indications for BCS [10, 21].

Radiotherapy is delayed until after delivery because of the high risk of foetal radiation exposure. In very rare instances where radiation treatment is indicated, medical physics and radiation expertise are required to limit foetal dose to the barest minimum. However, this is absolutely contraindicated with the Cobalt^60^ teletherapy (which is still available in many LMIC) due to the constant emission of background radiation [26]. Patient counselling on the risks and benefits of radiotherapy specific to their situation is mandatory.

Exposure to chemotherapeutic and systemic agents during the first trimester of pregnancy carries the greatest risk of congenital abnormalities, stillbirth and miscarriage [15, 16, 23, 27, 28]. The incidence of congenital malformations is reportedly low when chemotherapy is administered in the second or third trimester [15, 23, 27, 28]. Early second-trimester serial ultrasound assessment of foetal well-being, including foetal anomaly scan at 18–22 weeks gestation and serial growth scans from 26 weeks gestation, is important to monitor foetal health during chemotherapy [16]. Anthracycline-based chemotherapy is the most studied and therefore the safest option to date. Currently, there is insufficient evidence to inform the safe use of taxanes during pregnancy, whereas methotrexate, trastuzumab and tamoxifen are associated with foetal anomalies and therefore absolutely contraindicated [28]. Delaying the use of trastuzumab in Her2+ PABC even though unavoidable may negatively influence disease outcomes. Though uncommon, foetal growth restriction may occur during the use of some safe chemotherapeutic agents [27]. This could possibly explain the foetal growth retardation observed in our fourth case.

It is important to plan delivery following an adequate 3- to 4-week recovery from the transient reduction in maternal and foetal white blood cell and platelet counts to reduce the potential risk of infectious complications, bleeding and death that may occur from the last cycle of chemotherapy [9, 15, 16, 23, 27, 28]. The ultimate goal is to deliver at term, which is at least 37 weeks of gestation [15, 16]. When indicated, early delivery may be scheduled after attaining foetal lung maturity and assurance of care in an appropriate neonatal intensive care unit as in our first two cases [9, 15]. A study done in Ghana demonstrated that delivery after 36 weeks of gestation offers the best chance of survival [29]. In a few circumstances, early delivery is planned to avoid long breaks in treatment or to enable the start of a much-needed treatment if the patient is near term.

The mode of delivery is entirely based on obstetric indications [9, 16]. Two of our patients had an induction of labour with a normal outcome for the mother and the baby. Planned caesarean delivery may occur concurrently with mastectomy if deemed necessary, as was done for the second case.

Breastfeeding is contraindicated during radiation therapy, chemotherapy and hormonal therapies, requiring that patients must undergo counselling on limiting exposure to neonates, introduction to breastfeeding alternatives and coping strategies. Feeding from the treated breast after BCS is not contraindicated but may experience low volume milk production [9].

Long term follow-up is recommended for neonate and mother to monitor adverse effects on development that may occur and monitor for disease recurrence. A 30-year follow-up of children exposed to in-utero chemotherapy did not find any short or long term adverse effects and recommended full dosing of chemotherapy for pregnant women [30]. All female breast cancer survivors of childbearing age must undergo counselling on barrier methods of contraception to prevent new pregnancies which inadvertently leads to disruption of therapy and worse outcomes and increase in disease recurrence rates [9].

### Psychological support

The psychological burden of being confronted with a life-threatening disease and pregnancy is disconcerting. Careful breaking of bad news, trustworthy establishment of a patient-healthcare provider relationship, risk communication and guiding patients make an informed decision essential [31]. Inability to breastfeed or care for child interferes with mother–child bonding, compounded by fear of disease recurrence and partner abandonment worsens the psychological burden. Early psychological intervention, including counselling of close family and partner, improves coping skills and improves the quality of life.

## Conclusion

We have demonstrated from this case series that gestational breast cancer can be safely managed under multidisciplinary guidance in a resource-constrained country with good outcomes for mother and child, by following resource appropriate evidence-based practices. We recommend PABC in Africa should be referred to or supervised by hospitals with expertise and resources. Larger studies from Africa would determine the peculiar characteristics, cultural factors, prognostic features and outcomes of PABC. The limitation of our study is our small sample size.

## Conflict of interest

None.

## Funding

None.
